# Recent Advances in the Development and Antimicrobial Applications of Metal–Phenolic Networks

**DOI:** 10.1002/advs.202202684

**Published:** 2022-07-25

**Authors:** Yue Li, Yong Miao, Lunan Yang, Yitao Zhao, Keke Wu, Zhihui Lu, Zhiqi Hu, Jinshan Guo

**Affiliations:** ^1^ Department of Histology and Embryology School of Basic Medical Sciences Department of Plastic and Aesthetic Surgery Nanfang Hospital of Southern Medical University Southern Medical University Guangzhou 510515 P. R. China; ^2^ Regenerative Medicine and Tissue Repair Research Center Huangpu Institute of Materials Guangzhou 510530 P. R. China

**Keywords:** antimicrobial, coating, medical device modification, metal–phenolic networks (MPNs), wound healing

## Abstract

Due to the abuse of antibiotics and the emergence of multidrug resistant microorganisms, medical devices, and related biomaterials are at high risk of microbial infection during use, placing a heavy burden on patients and healthcare systems. Metal–phenolic networks (MPNs), an emerging organic–inorganic hybrid network system developed gradually in recent years, have exhibited excellent multifunctional properties such as anti‐inflammatory, antioxidant, and antibacterial properties by making use of the coordination between phenolic ligands and metal ions. Further, MPNs have received widespread attention in antimicrobial infections due to their facile synthesis process, excellent biocompatibility, and excellent antimicrobial properties brought about by polyphenols and metal ions. In this review, different categories of biomaterials based on MPNs (nanoparticles, coatings, capsules, hydrogels) and their fabrication strategies are summarized, and recent research advances in their antimicrobial applications in biomedical fields (e.g., skin repair, bone regeneration, medical devices, etc.) are highlighted.

## Graphical abstract

This review discusses the outstanding antibacterial performance of metal–phenolic networks (MPNs) as biomaterials in recent years. The main components of MPNs and different forms of MPNs biomaterials are mainly introduced, and the antibacterial applications of MPNs‐based biomaterials in skin repair, bone regeneration, biodevices, and other fields are summarized.

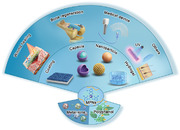

## 1 Introduction

Throughout human history, pathogenic microorganisms such as bacteria have always been an important factor in the morbidity and mortality of human beings.^[^ ^1^ ^]^ The discovery of antibiotics has provided a proven way to prevent and combat these microorganisms. However, the abuse of antibiotics and inadequate control of infections often lead to resistant and recurrent infections, as well as the associated complications, resulting in a major threat to public health globally.^[^ ^2^, ^3^ ^]^ According to Armentano and his colleagues, ≈2 million people in the United States, 33 000 people in the European Union, and more than 3 million people in developing countries die each year because of microbial infections caused by bacteria, including drug‐resistant bacteria.^[^ ^4^, ^5^ ^]^ The traditional antimicrobial agents used to fight bacterial infections are still dominated by antibiotics. However, due to the corresponding resistance mechanisms of bacteria, such as the production of passivating enzymes by bacteria, the acquisition and inheritance of drug resistance genes, the alteration of antibiotic binding sites, the permeability of cell membranes and active exocytosis mechanisms, as well as the formation of bacterial biofilms, antibiotics are currently encountering bottlenecks in the control of microorganisms.^[^ ^6^, ^7^, ^8^ ^]^ Therefore, scientists are increasingly inclined to develop novel antimicrobial agents to avoid the bacterial resistance and to exert functional properties that antibiotics do not possess in specific disease scenarios.

As the secondary metabolites found mainly in plants with phenol groups as the backbone structure, polyphenols are characterized by the presence of at least one or more phenolic rings in the structure and by the absence of nitrogen‐containing functional groups. Polyphenols have been intensively studied by scientists for their intrinsic anti‐inflammatory, antioxidant, antimicrobial, hemostatic, and disease healing properties.^[^ ^9^ ^]^ Furthermore, due to their favorable biocompatibility and chemically reactive phenolic hydroxyl groups that can chelate with metal ions, polyphenols often exert synergistic antibacterial effects with metal ions in MPNs.^[^ ^10^ ^]^ MPNs are supramolecular inorganic–organic hybrid networks formed on the basis of coordination between different metal ions and phenolic ligands, similar to crystalline porous coordination polymers (PCPs) and metal–organic frameworks (MOFs).^[^ ^11^ ^]^ The metal ions often used in MPNs, including Cu^2+^, Eu^3+^, Zn^2+^, Mg^2+^, and Ag^+^, also possess considerable antibacterial capabilities, and always have synergistic effects with polyphenols. Therefore, MPNs have been considered by many scientists as a category of powerful new antibacterial agents. On the other hand, metal ions also possess additional functionalities useful in the biomedical field. Cu^2+^ and Eu^3+^ are commonly reported metal ions could promote angiogenesis, while Zn^2+^ and Mg^2+^ are widely used in the field of orthopedic materials by promoting mineralization and regulating macrophage polarization, and Fe^3+^ is effective in the treatment of tumors and wounds due to its photothermal effect and mediated Fenton reaction.^[^ ^12^, ^13^, ^14^, ^15^, ^16^, ^17^, ^18^, ^19^, ^20^ ^]^ Compared to their counterparts, MPNs exhibit better properties: wherein, the inorganic material play a variety of roles in promoting tissue regeneration, enhancing photothermal effect, exerting antimicrobial properties, acting as an important component of a sensor, or providing specific magnetic, electronic or electrochemical properties.^[^ ^21^, ^22^, ^23^, ^24^, ^25^ ^]^ And the organic components greatly expand the range of available substrates by modifying material shape or material substrate properties, modifying hydrophobicity, providing active reaction interfaces, contributing their specific physicochemical properties such as electrical or optical properties or acting as important components in some electrochemical or biochemical reactions.^[^ ^23^, ^26^, ^27^ ^]^

MPNs, as amorphous networks, could be fabricated into various forms such as nanoparticles, hollow capsules, and hydrogels, and could be used as surface coating agents due to the high affinity providing by phenolic hydroxyl groups.^[^ ^28^, ^29^, ^30^, ^31^, ^32^, ^33^, ^34^, ^35^ ^]^ The coordinated self‐assembly between phenolic ligands and metal ions exploiting the unique properties of polyphenols and metal ions has a wide range of applications in the biomedical fields.^[^ ^36^, ^37^ ^]^ For example, epigallocatechin gallate (EGCG) and Mg^2+^ have been used to form composite coatings in situ on orthopedic titanium implants to enhance the osseointegration at the bone‐implant interface.^[^ ^38^ ^]^ The tetracycline hydrochloride loade poly(acrylic acid) (PAA) hydrogel/composite formed by the polymerization of acrylic acid initiated by the self‐catalytic Fe^3+^/TA‐cellulose nanofibers exhibited ultrashort gel time (≈30s) and favorable antibacterial ability, thus are promising to be applied in a wide range of biomedical fields.^[^ ^31^ ^]^ A MPN coordination gel based on natural low‐cost tannic acid (TA) and Ti^4+^ exhibited good in situ gelation property and the ability to bind other metal ions. Five metal ions, including Fe^3+^, Cu^2+^, Zn^2+^, Co^2+^, and Ni^2+^, were always incorporated into MPN systems to develop smart gel dressings for the treatment of infected wounds.^[^ ^39^ ^]^ However, the above examples can hardly encompass the applications in different biomedical areas of MPNs as a multifunctional platform.

To date, several reviews have been published on MPNs in interfacial modification or nanoparticle engineering.^[^ ^40^, ^41^, ^42^, ^43^ ^]^ However, these reviews mostly focus on the synthesis and fabrication of interfacial coatings and nanocomplexes of MPNs, and their applications are limited on drug delivery for tumor therapy and diagnosis. Few reviews, systematically summarized the antimicrobial applications of MPNs in the biomedical fields (e.g., in dermatology, orthopedics, or as medical devices, etc.). Therefore, in this review, we will summarize the multifaceted antimicrobial application potentials and mechanisms of MPNs in biomedical fields. We mainly develop three progressive themes, including the polyphenols and metal ions that constitute MPNs; the main biomaterial forms and fabrication approaches of MPNs; and the antimicrobial applications of MPN‐based biomaterial in different biomedical fields. Relevant research involves broad and in‐depth interdisciplinary issues, including not only materials science, surface engineering, and nano‐/microparticle technology, but also physiology and pathophysiology. We start with a brief introduction of polyphenols and metal ions commonly used in MPNs. Thereafter, we describe the fabrication approaches and different forms of MPNs‐based biomaterials, including nanoparticles, coatings, capsules, and hydrogels. Finally, we introduce the MPN‐derived antibacterial materials and highlight their antimicrobial applications in different biomedical fields (skin repair, photothermal antibacterial, bone regeneration, and anti‐infection coatings on biomedical devices). Overall, this review is expected to provide new insights and ideas for the design, preparation, and application of MPN‐based antimicrobial biomaterials, and facilitate their applications in a more broad field.

## 2 Polyphenols and Metals in MPNs

### 2.1 Polyphenols

As a class of naturally derived bioactive chemicals, polyphenols are abundantly found in plants (mainly fruits, vegetables, tea leaves, and roots).^[^ ^44^, ^45^ ^]^ As many as 8–9 thousands polyphenols have been extracted and identified, their chemical structures are therefore of great complexity and diversity (**Table** **1**).^[^ ^46^ ^]^ Usually these substances have one or multiple aromatic rings and with one or more hydroxyl groups attached to each aromatic group, a structural feature that also gives these compounds great biological activity. The mainstream classification of polyphenols is based on their basic chemical structure characteristics, and they are generally classified into five main categories: flavonoids, phenolic acids, stilbenes, lignans, and others.^[^ ^47^, ^48^ ^]^ The number of flavonoids and phenolic acids accounts for more than 90% of the total number of natural polyphenols. Due to their high reactivity and diverse structures, polyphenols exhibit anti‐inflammatory, antioxidant, antibacterial, antidiabetic, and hemostatic properties.^[^ ^44^, ^49^, ^50^, ^51^, ^52^, ^53^, ^54^, ^55^, ^56^ ^]^

**Table 1:** Chemical structure of typical polyphenols

Polyphenol	Structure	Polyphenol	Structure
Tannic acid	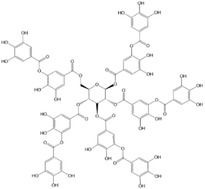	Catechin	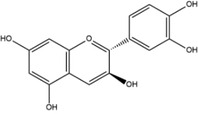
Epicatechin	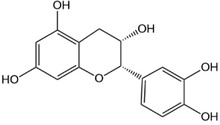	Epicatechin‐3‐gallate	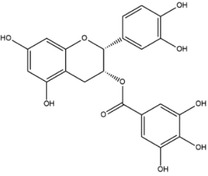
Epigallocatechin	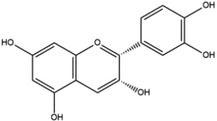	Epigallocatechin gallate	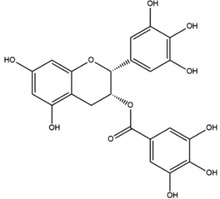
Gallic acid	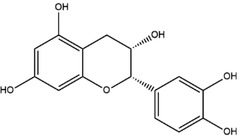	Ellagic acid	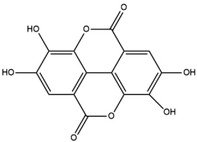
Caffeic acid	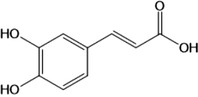	Protocatechuic aldehyde	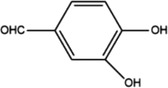
Resveratrol	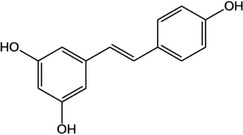	Quercetin	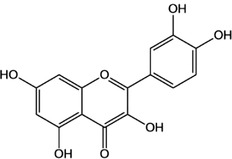
Catechol	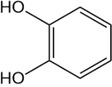	Pyrogallol	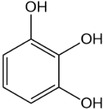

Most polyphenols have intrinsic antimicrobial activity. The antibacterial activity of polyphenols has been widely studied, the antibacterial ability of them was reported mainly related to their structure.^[^ ^57^, ^58^, ^59^, ^60^, ^61^, ^62^ ^]^ However, it is quite difficult to completely elucidate the antibacterial characteristics of different polyphenols due to their wide variety in structures. In general, the main possible mechanisms are as follows: 1) interaction with the bacterial cell wall/membrane; 2) inhibition of biofilm formation; 3) inhibition of bacterial enzymes and substrate deprivation; 4) protein regulation; 5) metal iron deprivation due to their metal‐chelating ability.^[^ ^58^, ^63^, ^64^, ^65^ ^]^ As a representative polyphenol approved by the U.S. Food and Drug Administration (FDA) for the use as a food additive, tannic acid (TA) exhibits favorable antimicrobial activity. Ramazani found that the activity of TA against *Staphylococcus aureus* (*S. aureus*) and *Escherichia coli* (*E. coli*) depended on the phenolic hydroxyl content, so higher TA concentration led to a stronger antibacterial effect and longer duration of antibacterial activity.^[^ ^66^ ^]^ Gallic acid (GA) is one of the hydrolysis products of TA, which produces glucose and multiple GA molecules upon hydrolysis.^[^ ^67^ ^]^ Simões and co‐workers deemed that the inhibition ability of GA against *E. coli*, *P. aeruginosa*, *S. aureus* potentially caused by the irreversible changes of the bacterial cell membrane (charge, internal and external permeability, and physicochemical properties) by altering hydrophobicity, reducing negative surface charge, and causing localized rupture and perforation of the bacterial cell membrane, subsequently leading to leakage of substances necessary for bacterial survival from the bacterial cell.^[^ ^68^ ^]^ Most commonly used polyphenols, such as tea polyphenols (TPs), anthocyanins (ACs), caffeic acid (CA), and catechins (Cat), have similar antimicrobial mechanisms and properties as mentioned above.

**Figure 1:**
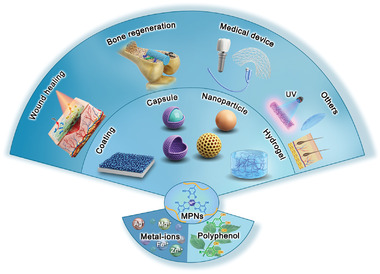
The fabrication of metal–phenolic networks (MPNs), different forms of MPNs, and the antimicrobial applications of MPNs in various biomedical fields.

Polyphenols show great biological activity and chemical reactivity due to their multiple polyphenolic hydroxyl structure can react with various organic or inorganic substances. Their common reaction/interaction modes are: 1) hydrogen bonding; 2) electrostatic interactions; 3) hydrophobic interactions; 4) metal bonding; 5) covalent bonding; 6) *π*–*π* stacking.^[^ ^69^ ^]^ Among these polyphenol‐mediated interactions, the coordination of polyphenols with metal ions is our main concern in this review. **Figure** **1**

### 2.2 Metal Ions

**Figure 2:**
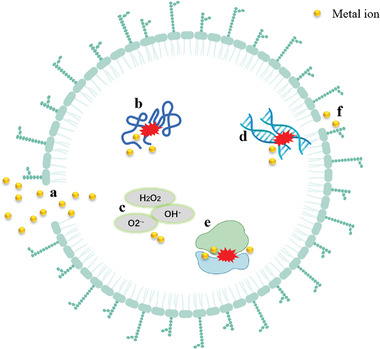
A model demonstrating the potential antibacterial mechanisms of metal ion: a) Breakage of cell membranes leading to leakage of intracellular material. b) Interaction with proteins. c) Reactive oxygen species (ROS) production. d) Interaction with DNA. e) Inhibition of ribosome function leading to mis‐synthesis of proteins; and f) membrane pits that appear upon exposure to metal ion.^[^ ^11^ ^]^

Metals (such as Ag, Au, Fe, Cu, Zn, Ca, Ti, Co, V, Zr, La, Gd, and Tb) have been used in different material forms as antimicrobials for centuries. The metal ions released from metal‐containing compounds can well inhibit the growth of microbes. Noble metals (Ag, Au) are widely used in antimicrobial scenarios, and they are extremely poisonous to most bacteria and have antimicrobial and bactericidal activity at exceptionally low concentrations, and they have also been widely reported to have photothermal antibacterial activity.^[^ ^70^, ^71^, ^72^ ^]^ Further, some transition metal ions (Fe^3+^, Cu^2+^, Zn^2+^, Ca^2+^, Ti^4+^, Co^2+^, V^3+^, Zr^4+^) also play a conventional broad‐spectrum antibacterial effect.^[^ ^73^ ^]^ Some of these metal ions (Fe^3+^, Cu^2+^, Zn^2+^, Ca^2+^) are also common trace elements in the human body and play an important physiological role. Iron is mostly found in hemoglobin, ferritin, and enzymes related to hematopoiesis, and is an important building material for the blood and hematopoietic system.^[^ ^74^ ^]^ Copper ions have been reported to have an important physiological role in antivascular thrombosis and in promoting wound healing through the continuous catalytic breakdown of endogenous S‐nitrothiols (RSNOS) and in situ release of nitric oxide in a controllable manner, in addition to its involvement in hematopoietic processes and as an essential component found in several active enzymes.^[^ ^75^, ^76^ ^]^ Zinc ions mostly exist in skeletal muscle, bone, liver, and skin. Zinc is a co‐catalyzer for many enzymes and an essential component of many transcription factors, and is also involved in the synthesis of many transporter proteins and zinc finger proteins.^[^ ^77^ ^]^ Calcium ions are also very important in the human body and 99% of the body's calcium is involved in the composition of bones and teeth.^[^ ^78^ ^]^ Some rare earth ions have also been found to have good antibacterial effects. In Shi's work, a Cat‐Re^3+^ MPN films formed by the self‐assembly of several Re^3+^ (La^3+^, Gd^3+^, Tb^3+^) with catalyzer was found to have good and long‐term antibacterial efficiency (>90%) against *P. aeruginosa*.^[^ ^79^ ^]^ Although the antibacterial mechanisms of these metal ions and the corresponding metal compounds have not been fully explored. The widely acceptable antimicrobial mechanisms include: 1) interaction of metal ions with the bacterial cell membrane; 2) disruption of the bacterial cell membrane and leakage of intracellular material due to metal ions; 3) excitation of reactive oxygen species (ROS) by metal ions resulting in bacteria damage; 4) binding of metal ions to related proteins, peptides, DNA and other substances, which can damage such substances and affect their function (**Figure** **2**).^[^ ^80^, ^81^, ^82^ ^]^

### 2.3 MPNs

Compared to metals and metal ions alone, MPNs have many advantages and good adaptability to various microenvironments. Similar to mussel‐inspired catechol groups, polyphenols are rich in phenolic hydroxyl groups and have good adhesion properties to various surfaces.^[^ ^40^, ^83^ ^]^ Therefore, polyphenols are enable to serve as an important mediator to introduce metal ions onto biomaterial surfaces to exert their biological effects. MPNs are always pH‐responsive, the interaction between polyphenols and metal ions is easier to be dissociated at low pH,^[^ ^37^, ^84^, ^85^ ^]^ conferring MPNs with pH‐adjusted metal ion release ability beneficial for different application scenarios. Most polyphenols have strong antioxidant properties, which can effectively scavenge excess ROS induced by metal ions and maintain the homeostasis of the local microenvironmental.^[^ ^86^, ^87^ ^]^ Polyphenols can also synergize with metal ions to enhance the antibacterial activity.^[^ ^71^ ^]^

## 3 Fabrication of MPNs‐Based Materials

The preparation of MPNs is usually facile and efficient. Through a simple physical mix, the positively charged metal ions spontaneously chelate with the electron‐dense phenolic hydroxyl groups of the polyphenols (the degree of chelation is mostly dependent on temperature, pH, etc.), which often react spontaneously to form particles with varying sizes (nano to micrometer scale), and if templates with different shapes (e.g., flat, spherical, and irregular shapes) were provided, the MPNs can be well deposited to form coatings, films, and capsules depending on the shapes (**Table** **2**). In addition, polyphenols are often incorporated into hydrogel systems because of their abundant phenolic hydroxyl groups, which also allow them to react with other substances even after coordination with metal ions.

**Table 2:** Different structures of MPNs

Phenolic ligands	Metal ion	Structure of MPNs	Refs.
EGCG	Ag^+^	Nanoparticles	[95]
PACs	Ti^4+^	Nanoparticles	[90]
TA	Ag^+^	Nanoparticles	[88]
TA	Fe^3+^	Nanoparticles	[89]
TPs	Ag^+^	Nanoparticles	[94]
TA	Cu^2+^	Coating	[98]
TA	Ag^+^	Coating	[96]
Cat	La^3+^, Gd^3+^, Yb^3+^	Coating	[79]
TA, GA, PC, PA	Fe^2+^, Fe^3+^, Cu^2+^, Co^2+^, V^3+^	Coating	[102]
PEG‐polyphenol	Fe^3+^	Capsule	[106]
TA	Al^3+^	Capsule	[110]
EA, TA	Zn^2+^	Capsule	[112]
EGCG, ECG, EGC, EC	Fe^3+^	Capsule	[113]
TA	Fe^3+^, Ti^4+^, Zr^4+^	Hydrogel	[124]
TA	Ag^+^	Hydrogel	[119]
TA	Ti^4+^	Hydrogel	[117]
TA	Fe^3+^	Hydrogel	[129]

### 3.1 MPN Nanoparticles

**Figure 3:**
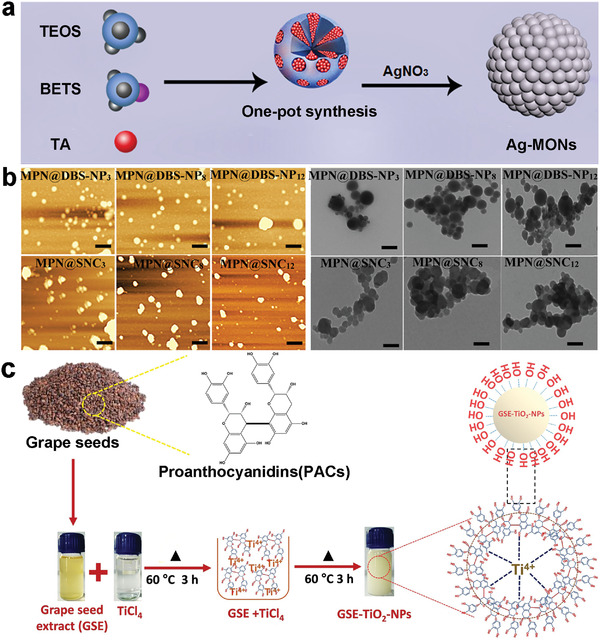
a) Representation of one‐pot synthesis of Ag‐mesoporous organosilica nanoparticles (MONs). Reproduced with permission.^[^ ^88^ ^]^ Copyright 2020, American Chemical Society. b) AFM and TEM images of MPN@debranched starch nanoparticles (DBS‐NP) and MPN@starch nanocrystals (SNC) systems. Reproduced with permission.^[^ ^89^ ^]^ Copyright 2019, American Chemical Society. C) Simple schematic diagram of the synthesis of Grape seed extract (GSE)‐TiO2‐NPs. Reproduced with permission.^[^ ^90^ ^]^ Copyright 2021, Wiley‐VCH.

Using the coordination of metal ions with polyphenols, a variety of MPN nanoparticles could be synthesized through a facile one‐step procedure, and other substances could also be composed to confer them of different properties.^[^ ^91^, ^92^, ^93^ ^]^ Silver ions are one of the most common metal ions used in antimicrobial MPN nanoparticles, which are often in situ reduced from silver ion by polyphenols serving as reducing agents, stabilizers, and efficient antioxidants. For example, in Dan's work, TA and silver ions were deposited onto degradable silica nanoparticles through a facile one‐step procedure in the presence of glutathione (GSH). The silver ions were irreversibly released as the complex matrix degraded, exhibiting excellent antibacterial properties against *S. aureus* and *E. coli* (**Figure** **3**a).^[^ ^88^ ^]^ Similarly, tea polyphenols (TPs), an extract of green tea, were also used to reduce silver ions into silver nanoparticles with excellent antimicrobial properties, and the outermost layer was coated with polyethylene glycol (PEG) to enhance the dispersibility and biocompatibility of the particles.^[^ ^94^ ^]^ In addition, polyphenols such as catechins and EGCG can stabilize the reduced silver nanoparticles to produce multifunctional nanoparticles with superior antibacterial, anti‐cancer and antioxidant properties.^[^ ^95^ ^]^ Jin and his colleagues encapsulated TA and iron ions on starch nanoparticles/nanocrystals to form composite nanoparticles with effective antibacterial and antioxidant properties. The nanoparticles exhibited pH‐responsive release due to that Fe^3+^ and polyphenol phenolic hydroxyl groups are more stable under alkaline conditions and easily dissociated under acidic conditions (Figure 3b).^[^ ^89^ ^]^ Alomary performed a one‐step synthesis of proanthocyanidins (derived from grape seed extract) with Ti^4+^, and the resulting TiO4 nanoparticles attached with polyphenols showed significant inhibitory effects on pathogens associated with urinary tract infections (Figure 3c).^[^ ^90^ ^]^

### 3.2 MPN Coatings

**Figure 4:**
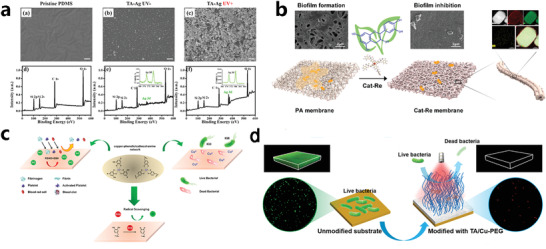
a) SEM images and XPS spectra demonstrate the accelerated deposition of TA–Ag nanoparticles on PDMS assistant with UV light irradiation. Reproduced with permission.^[^ ^96^ ^]^ Copyright 2021, American Chemical Society. B) Assembly and SEM mapping images of the composite of catechins and rare‐earth ions on porous polyamide membranes. Reproduced with permission.^[^ ^79^ ^]^ Copyright 2020, American Chemical Society. C) Preparation of copper‐catechin‐based coatings.^[^ ^97^ ^]^ Copyright 2018, American Chemical Society. D) Modification of TA/Cu‐PEG composite coating. Reproduced with permission.^[^ ^98^ ^]^ Copyright 2021, American Chemical Society.

The MPN system has been widely used as thin films and coatings, to confer antibacterial or anti‐inflammatory activities, harmful substances filtration, or water–oil separation ability, improve biocompatibility, or as drug carriers and even dyeing of wigs.^[^ ^79^, ^96^, ^99^, ^100^, ^101^, ^102^, ^103^ ^]^ The gallol groups on natural polyphenols can chelate with a variety of metal ions to form MPNs, and the multihydroxyl structure serving as hydrogen bonding acceptors and donors, enabling polyphenols to interact with a wide range of bioactive substances and also to be easily coated on various hydrophilic and hydrophobic surfaces.^[^ ^40^, ^101^, ^104^, ^105^ ^]^ Thus‐obtained MPN coatings often have excellent antimicrobial performance. For instance, the accelerated deposition of TA‐AgNPs nanocomposite coating on the surface of dimethyl siloxane (PDMS) or other substrates under UV light brought significant antimicrobial activity in vivo, suggesting a potential mean for antimicrobial applications (**Figure** **4**a). Shi and his co‐worker self‐assembled catechin with rare‐earth ions (La^3+^, Gd^3+^, Yb^3+^) on the surface of polyamide (PA) membranes, synergistically prevented the adhesion of *P. aeruginosa* and the subsequent biofilm formation (Figure 4b).^[^ ^79^ ^]^ Similarly, Cu^2+^ has been reported could chelate with TA or polypamine, resulting in a MPN coating with antimicrobial ability, superior antioxidant properties, and anti‐inflammatory properties, as well as excellent biocompatiblity beneficial for blood contact biomedical devices (Figure 4c).^[^ ^97^ ^]^ In Yu's work, they also utilized TA for rapid chelation with Cu^2+^ to form a TA/Cu‐PEG composite membrane together with PEG. This composite membrane has photothermal antibacterial property, bacterial adhesion prevention ability, and good biocompatibility, which is effective in preventing biofilm formation and solving the associated infection problems in biomedical materials and devices (Figure 4d).^[^ ^98^ ^]^

### 3.3 MPN Films and Capsules

**Figure 5:**
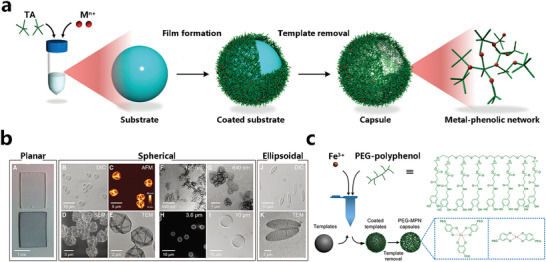
a) Assembly of TA and different metal ions on sphere templates followed template removal resulted in MPN capsules. Reproduced with permission.^[^ ^37^ ^]^ Copyright 2014, Wiley‐VCH. B) Planar deposition of MPNs and SEM, AFM, TEM imaging of spherical and ellipsoidal MPN capsules.^[^ ^36^ ^]^ c) Rapid assembly of Fe^3+^ with PEG‐polyphenol on a sphere template followed by template removal to give PEG‐MPN capsules. Reproduced with permission.^[^ ^106^ ^]^ Copyright 2015, American Chemical Society.

As a system combining inorganic and organic components, MPNs can be assembled into hollow capsules with a series favorable properties, including selective permeability, considerable mechanical/thermal stability, and stimuli responsiveness.^[^ ^107^, ^108^, ^109^, ^110^, ^111^ ^]^ The properties of MPN capsules including membrane thickness, disassembly properties, and fluorescence behavior are mostly determined by the coordinated metal ions.^[^ ^112^, ^113^, ^114^, ^115^, ^116^ ^]^ In addition, many functional polyphenol derivatives introduced by combining natural polyphenol ligands with (bio)macromolecules have been extensively investigated. The preparation of MPN capsules can be easily achieved by facile deposition of MPN coating on the surface of a template followed by removing of the substrate template (**Figure** **5**a).^[^ ^37^ ^]^ Caruso and his colleagues obtained flat films, spherical capsules as well as ellipsoidal capsules using TA and Fe^3+^ depositing on templates with different shapes (Figure 5b). Interestingly, they found that the stoichiometric ratio adjustment can affect the thickness of the film as well as the surface roughness, and the concentration of Fe^3+^ is the main factor affecting the thickness of the capsule or film when the amount of TA is relatively higher than Fe^3+^. At the same time, this MPN capsule showed pH‐dependent decomposition, they were apt to dissociate in more acidic microenvironments. The color of the capsule suspension is also PH‐dependent, with the suspension being colorless at pH < 2, blue at 3 < pH < 6, and red at pH > 7.^[^ ^36^ ^]^ To address the poor water solubility of dietary flavonoids (Myricetin (Myr), quercetin (Que), fisetin (Fis), and luteolin (Lut)), Caruso also ligated these flavonoids with metal ions and assembled them into capsules and films, making them more water‐soluble, while still possessing superior antioxidant activity and free radical scavenging ability, with future potential as cytoprotective and inflammation scavenging coatings. Overall, the chemical network enables aqueous deposition on planar and particle templates with a fast and easy assembly process at low cost, coupled with pH responsiveness and negligible cytotoxicity.

A series of multifunctional MPN capsules have been extensively studied and developed. For instance, PEG‐polyphenols were used for the assembly of coordination complexes with Fe^3+^, which exhibited rapid assembly, low contamination, and pH‐compatible properties. Compared to TA‐MPN capsules, PEG‐MPN capsules exhibit reduced nonspecific protein adsorption and cell binding, as well as a faster degradation rates under specific pH values (Figure 5c). Another drug sustained release capsule with pH responsiveness is a simple mixture of polystyrene sulfonate (PSS) doped calcium carbonate particles with adriamycin (DOX) followed by deposition of MPN ligand films of TA and Al^3+^ around the composite template to obtain monodisperse DOX‐loaded MPN capsules.^[^ ^110^ ^]^ Hollow capsules composed of GA/Fe^3+^ were found to possess redox activity and controlled “on‐off” regulatory properties and could be used for electrochemical studies.^[^ ^114^ ^]^ Based on the synergistic interaction of metal–organic frameworks (MOFs) with natural polyphenols under weak alkaline conditions, this simple strategy enables the direct fabrication of various phenolic functional materials or metal–phenol frameworks (MPFs) with controlled hollow nanostructures (polyhedra core–shell, corrugated, hollow cage, etc.) and controlled size, morphology, roughness and composition, such as Zn‐EA hollow cages, Fe‐EA polyhedra, PB‐based MPF, and Au@Co‐MPF.^[^ ^112^ ^]^

### 3.4 MPN Hydrogel

**Figure 6:**
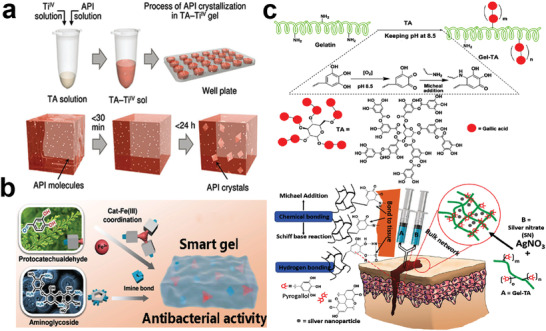
a) Spontaneous gel formation of TA and Ti^4+^ and drug active ingredients. Reproduced with permission.^[^ ^117^ ^]^ Copyright 2018, Wiley‐VCH. B) Smart hydrogels with dynamic covalent bonds prepared from protocatechualdehyde, Fe^3+^, and aminoglycosides. Reproduced with permission.^[^ ^118^ ^]^ Copyright 2019, American Chemical Society. C) Synthesis of sliver nitrate crosslinked tannic acid modified gelatin (Gel‐TA‐SN) hydrogels for tissue repair. Reproduced with permission.^[^ ^119^ ^]^ Copyright 2018, Elsevier.

Hydrogels are gels with a 3D network structure, have attracted great interest of biomaterial scientists for many years and are used in a wide range of applications due to their hydrophilic and biocompatible properties.^[^ ^120^, ^121^, ^122^ ^]^ The multihydroxyl structures of polyphenols allow them to cross‐link with other substances through covalent or noncolvent bonds, such as chelating with metal ions or forming dynamic ester bonds.^[^ ^119^, ^123^ ^]^ Among these polyphenols, TA is considered to be the best choice for the preparation of gels due to its high density hydroxyl structure and good water solubility.^[^ ^105^ ^]^ For example, the appropriate ratio of TA and Ti^4+^ in *N*,*N*‐dimethylformamide (DMF), *N*‐methyl‐2‐pyrrolidone (NMP), and aqueous solution can form MPN gels directly. However, the direct in situ gelation was not observed for other metal ions such as Fe^3+^ or other transition metal ions, suggesting that this phenomenon is unique for group IVB metal elements.^[^ ^124^ ^]^ Meanwhile, the same supramolecular MPN gels formed by TA and Ti^4+^ were used as a medium for the crystallization of active pharmaceutical ingredients (API) (**Figure** **6**a). These gel‐API crystalline composites can continuously release drug, indicating a great potential as drug carrier.^[^ ^117^ ^]^ An adsorption system with a high affinity to various metal ions formed by cross‐linking of TA and Zr^4+^, could spontaneously gel within 3 min. The system was optimized by adjusting the polyphenol/metal ion ratio to 1:1.2 so that most of the chelating sites of tannins in the system could be used to capture other metal ions. In addition, the system exhibited good pH and thermal stability, and has the best metal ion adsorption and removal efficiency at pH 5.^[^ ^125^ ^]^ Interestingly, for the above types of MPN gels, Steven and his colleagues found that these MPN gels exhibited excellent biocompatibility and low immunogenicity in vivo.^[^ ^126^ ^]^ Furthermore, MPN hydrogels showed longer drug sustained‐release time (from <1 to 10 days) comparing with Pluronic F127 hydrogels commonly used in vivo.^[^ ^127^, ^128^ ^]^

Some smart responsive MPN gels have also been intensively investigated. TA/Fe^3+^ was incorporated into poly(*N*‐isopropyl acrylamide) (PNIPAAm) hydrogels as a photothermal sensor. Where TA is linked to PNIPAAm by hydrogen bonding during in situ polymerization through its abundant pyrogallol and catechol groups. TA and Fe^3+^ enhanced the mechanical properties of the hydrogel while conferring excellent photothermal properties as well as reproducible deformation behavior upon near‐infrared (NIR) irradiation.^[^ ^129^ ^]^ Another smart hydrogel formed by the combination of protocatechualdehyde (Cat) and Fe^3+^ exhibited excellent dynamic properties and multiresponsiveness to different stimuli (temperature, light, pH, electricity, and redox), as well as a good antibacterial activity (Figure 6b).^[^ ^118^ ^]^ Some hydrogels play an important role in tissue repair by close wounds, sealing tissues, and providing antimicrobial and hemostatic activities. Guo reported a bioadhesive formed through a simple one‐step Michael addition of TA and gelatin under oxidizing conditions, followed by further cross‐linking by silver nitrate, to give in situ reduced silver nanoparticles loaded bioadhesive possessing that excellent antimicrobial ability (Figure 6c).^[^ ^119^ ^]^

## 4 Antibacterial Application of MPNs‐Based Biomaterials

Two is better than one is an opposite description of MPNs. Compared to metal ions and phenolic substances, their dynamic interactions give MPNs additional advantages and properties: 1) the polyhydroxy structure of polyphenols can chelate metal ions to form a stable chemical network; 2) the structure formed can provide a slow release of antibacterial metal ions (and therefore prolong the duration of antibacterial activity); 3) polyphenols can reduce the cytotoxicity of metal ions; 4) polyphenols can reduce the level of reactive oxygen species (ROS) provoked by metal ions, protecting cells, and surrounding tissues; 5) polyphenols can play a synergistic antibacterial role with metal ions; 6) some MPNs have unique photothermal antibacterial effects, etc. MPNs presenting different material morphologies (hydrogels, dressings, coatings, scaffolds, etc.) are widely used in biomedical fields such as skin wound healing and regeneration, bone tissue repair, medical implants, and electronic smart tissue engineering (**Table** **3**).

**Table 3:** The antimicrobial applications of different forms of MPN‐based biomaterials

Phenolic ligands	Metal ion	Structure of MPNs	Bacteria	Application	Refs.
PAs	Cu^2+^	Ch/PAs‐Cu Sponge Dressing	*S. aureus, E. coli*	Wound healing	[140]
EGCG	Ag^+^	HG‐Ag‐EGCG Hydrogel patches	*S. aureus, E. coli., P. seudomonas, B. subtilis*	Wound healing	[143]
TA	Zn^2+^	CTZ1‐4 Hydrogels	*E. coli*	Wound healing	[150]
Lignin	Ag^+^	NPs‐P‐PAA hydrogel	*S. epidermidis, E. coli*	Wound healing	[131]
Cat	Fe^3+^	LFe‐CMC‐MC Hydrogels	*S. aureus*	Wound healing	[151]
EGCG	Mg^2+^	Mg/EGCG/ HTCC‐Ce6 Nanoparticles	*S. aureus, E. coli*	Wound healing	[155]
TA	Ag^+^	STA hydrogel	*S. aureus, E. coli*	Infected wound healing	[130]
PA	Fe^3+^	QCS‐PA@Fe Hydrogel	*S. aureus, E. coli, MRSA*	Infected wound healing	[137]
TA	Fe^3+^	HPCH/TA/Fe hydrogels	*S. aureus, E. coli*	Infected wound healing	[142]
TA	Fe^3+^	MoS2@TA/Fe NSs Hydrogel	*S. aureus, E. coli*	Infected wound healing	[153]
TA	Fe^3+^	HPCH/TA/Fe Hydrogel	*S. aureus, E. coli*	Infected wound healing	[136]
TA	Fe^3+^	ATF hydrogels	*S. aureus*	Infected wound healing	[154]
Qr	Ti^4+^	TiO2@UCN/Qr/LA Coating	*S.aureus*	Bone regeneration	[161]
TA	Ag^+^	PU/AgTHA Microparticulates	*S. aureus*	Orthopaedic antibacterial implant	[159]
TA	Ag^+^	Citrate‐based tannin‐bridged bone composite	*S. aureus, E. coli*	Lumbar fusion	[160]
TA	Fe^3+^	TBA‐Ag coating	*S. aureus*	Catheter‐associated infection	[172]
GA	Cu^2+^	CuII‐GA/CySA Coating	*S. aureus, E. coli*	Medical device anti‐infection	[171]
PG	Ag^+^, Mg^2+^	pPG/Ag/Mg Coating	*MRSA*	Catheter‐associated infections	[175]
Lignin	Fe^3+^	Fe‐SL‐g‐PAA Hydrogel	—	Human–machine electronics	[180]

A large number of MPNs have been reported to show excellent antibacterial properties. At present, the use of MPNs mainly depends on their application scenarios. For example, Fe^3+^ can be incorporated to confer the MPNs with favorable photothermal properties, Cu^2+^ can be used to promote angiogenesis, Zn^2+^ can be used to promote bone regeneration, and Ca^2+^ can be used to accelerate blood clotting. For polyphenols, more abundant phenolic hydroxyl groups tend to result in better performance of MPNs. We believe that along with more and more systematic research on MPNs emerging, the design principles and modification approaches of MPNs will be gradually clarified in the near future. The recent research progress of the antimicrobial applications of MPNs in different biomedical fields are listed below.

### 4.1 Skin Repair

#### 4.1.1 Wound Healing and Skin Regeneration

Today, acute and chronic wounds caused by trauma and many different diseases is a significant healthcare challenge. Wound healing is essentially a series of pathophysiological processes of tissue loss due to the action of damage‐causing factors, followed by local tissue repair, reconstruction, and regeneration.^[^ ^133^ ^]^ There are various types of wounds, namely acute wounds, chronic wounds, infected wounds, etc. And various types of biomaterials are used in wound healing, including dressings, hydrogels, tissue adhesives, etc.^[^ ^134^ ^]^ For the pathophysiological characteristics of wound healing, the ideal biomaterials for wound healing should have the characteristics of nontoxicity, prevention of bacterial infection, strong adhesion, wetting, absorption of excess exudate at the wound, good physical and mechanical properties, and low cost.^[^ ^134^ ^]^ In particular, it is important to emphasize that the problem of prolonged wound healing due to infection by harmful bacteria occupies the dominant position in wounds. White et al. analyzed the microorganisms of more than 2000 chronic wounds by 16S rRNA sequencing technology, and bacteria such as *S. aureus* and *P. aeruginosa* were the main species.^[^ ^135^ ^]^ MPNs with excellent antimicrobial properties can meet most of the requirements for biomaterials in trauma applications and is undoubtedly an excellent candidate in this regard.

**Figure 7:**
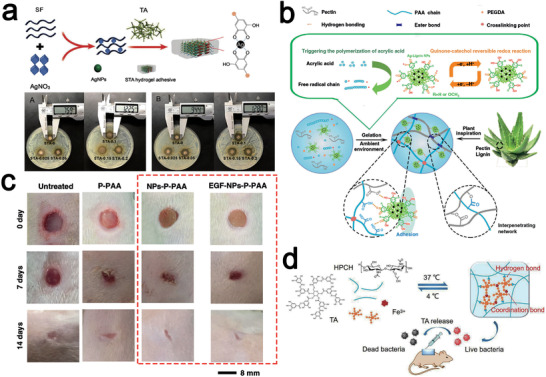
a) Schematic diagram of the preparation of (Silk Tannic Acid) STA hydrogel and its antibacterial performance. Reproduced with permission.^[^ ^130^ ^]^ Copyright 2020, Royal Society of Chemistry. B) Design strategy for the plant‐inspired tough, self‐adhesive, and antibacterial Ag‐Lignin NPs‐pectin‐polyacrylic acid (NPs‐P‐PAA) hydrogel. c) Therapeutic effect of NPs‐P‐PAA hydrogel on rat wounds. b,c) Reproduced under the terms of a Creative Commons Attribution 4.0 International License.^[^ ^131^ ^]^ Copyright 2019, The Authors, published by Springer Nature. d) Preparation of a temperature and pH‐sensitive hydroxypropyl chitin (HPCH)/TA/Fe composite hydrogel against microbial infections. Reproduced with permission.^[^ ^132^ ^]^ Copyright 2020, Elsevier.

MPNs are often used in combination with various other components such as chitin, sodium alginate, chitosan, silk fibroin, and polyacrylamide to optimize their functional properties.^[^ ^130^, ^136^, ^137^, ^138^, ^139^, ^140^, ^141^, ^142^ ^]^ For example, a bioadhesive based on silk fibroin (SF), TA loading with silver nanoparticles was developed, in which TA can both spontaneously interact with SF to form an adhesive matrix network and reduce silver ions in situ to generate silver nanoparticles, which has high wet adhesion strength, good self‐healing ability, and cytocompatibility. And when the silver nitrate concentration is greater than 0.05 wt%, the prepared silk tannic acid (STA) bioadhesive has good in vitro antibacterial properties and has a high potential for application in infected wound healing (**Figure** **7**a).^[^ ^130^ ^]^ Similarly, plant‐inspired adhesive hydrogel containing lignin, silver nanoparticles, pectin, and pectin acrylic acid (AA) formed a multicrosslinked interpenetrating network of covalent and noncovalent bonds conferring excellent mechanical properties to the hydrogel, while the lignin–silver nanoparticles construct a dynamic and durable catechol redox system that continuously generates catechol groups that can provide durable adhesion to this gel (Figure 7b). In addition, the adhesive hydrogel exhibited excellent toughness and antibacterial activity, while the NPs‐P‐PAA hydrogel‐treated tissue regenerated well in the Sprague Dawley (SD) rat full skin wound model, with ordered collagen fibers and regenerated hair follicles (Figure 7c).^[^ ^130^, ^131^ ^]^ Zhu and co‐workers prepared a cellulose crystal (CNC)‐Ag@AgCl nanocomposite with excellent toughness and good antibacterial properties by a simple one‐step procedure and stabilized in the network of sodium alginate hydrogel with the help of TA, which has potential applications in infected wound healing.^[^ ^141^ ^]^ In addition, hydrogel‐Ag‐EGCG (HG‐Ag‐EGCG) hydrogel patches composed of EGCG and silver nanoparticles, CG‐Ag‐Q wound dressings formed by PODA/AgNPs/quercetin doped in PDMS, and bimetallic Au@AgNPs with surface modification of various polyphenols have been developed for the healing of infected or noninfected wounds and have shown remarkable antimicrobial performance.^[^ ^143^, ^144^, ^145^ ^]^

From the above series of work on the application of MPNs in wound repair, it can be seen that silver is among one of the most widely used metal ions due to its antibacterial, antifungal, and antiviral activities. Silver is known to have a general antibacterial effect against Gram‐positive and Gram‐negative bacteria, such as *S. aureus*, *E. coli*., *P. aeruginosa*, *Streptococcus*, *Aspergillus*, and many drug‐resistant bacteria such as methicillin‐resistant *S. aureus* (*MRSA*), erythromycin‐resistant *Streptococcus*, and ampicillin‐resistant *E. coli*. Antimicrobial agents and biomaterials designed based on silver are widely used, and microorganisms are less capable of developing resistance to them. Silver ions or silver nanoparticles have long been one of the most commonly used nanomaterials in healthcare systems.^[^ ^146^ ^]^ The antibacterial mechanism of silver has been widely studied by scientists, and currently, there are several antibacterial mechanisms as follows: 1) silver nanoparticles and silver ions both act on the disulfide bonds of bacterial proteins and interact with the protein structure, making the bacteria dysfunctional; 2) silver ions can inhibit the replication and proliferation of bacteria by binding to bacterial DNA and denaturing it, where silver ions can bind to sulfhydryl proteins, leading to condensation and denaturation of DNA, which eventually leads to bacterial death; 3) silver ions are positively charged and the bacterial surface is negatively charged, resulting in electrostatic attraction between the two and subsequent deposition of silver on the bacterial surface; 4) due to their small particle size, silver nanoparticles are able to enter the bacterial cell, resulting in intracellular nanoparticle deposition that affects bacterial function; 5) silver nanoparticles can generate ROS and free radicals to damage bacteria; 6) silver nanoparticles can disrupt the bacterial cell wall leading to leakage of bacterial intracellular contents and affecting bacterial homeostasis.^[^ ^146^ ^]^ In addition, there are some potential mechanisms such as the effect of silver on some proton pumps and membrane proteins on the surface of bacterial cytosol and the loss of glutathione have also been reported.^[^ ^147^, ^148^, ^149^ ^]^ In conclusion, natural polyphenols of plant origin are used as a natural, inexpensive, convenient, and eco‐friendly biological reducing agent for the synthesis of silver nanoparticles at room temperature, and the MPN antibacterial system composed of both is very simple, economical, and green‐friendly for the application in the field of wound healing.

Besides silver ions, various metal ions such as copper, iron, and zinc ions have their corresponding antimicrobial effects and are used as a component of MPN systems and in combination with other substances for wound healing applications. For instance, a traumatic dressing prepared by surface modification of chitin fibers using proanthocyanidins (PAs) ligated with Cu^2+^ were shown to have antibacterial, antioxidant, and proangiogenic effects. On the one hand, PAs conferred antioxidant activity to chitin sponges. On the other hand, with the aid of PAs, Cu^2+^ could effectively catalyze S‐nitrosothiols (RSNOs) and sustain the production of nitric oxide (NO) in vitro and in vivo, which enhanced the proangiogenic activity and antibacterial activity. At the same time, the wound was shown to effectively promote cell proliferation and expression of related vascular growth factors such as vascular endothelial growth factor (VEGF), fibroblast growth factor (FGF), and matrix metalloproteinase 9 (MMP9) and regulate the level of inflammation in the wound, ultimately promoting wound healing and inhibiting scar formation.^[^ ^140^ ^]^ In contrast, a thermosetting and Ph‐responsive hydrogel constructed from TA, Zn^2+^, and carboxy agarose was found to inhibit NO production by macrophages to suppress inflammation levels, and it also proved to have excellent antimicrobial properties due to the presence of TA as well as Zn^2+^, with practical translational implications in the trauma field.^[^ ^150^ ^]^ Due to the complexity of the wound microenvironment, the preparation of gels or dressings that are responsive to conditional stimuli (temperature, pH, light, etc.) is of particular importance. A hydroxypropyl chitin (HPCH)/TA/Fe composite hydrogel composed of hydroxypropyl chitin, TA and iron ion, which is also pH‐responsive and thermosensitive, is capable of rapid gelation at physiological temperature, which means that the precursor solution of this hydrogel can be injected onto arbitrarily irregularly shaped wound areas and perfectly form a gel to cover the wounds (Figure 7d). In the low pH microenvironment of the wound, the MPNs of TA and Fe^3+^ within the hydrogel slowly dissociates, releasing TA and Fe^3+^ with broad‐spectrum and long‐lasting antibacterial activity (7 days).^[^ ^136^ ^]^ Lee reported another Fe^3+^‐containing metallo‐polyphenol blue cross‐linked hydrogel, which is mainly composed of two different chitosan (catechol modified methacryloyl chitosan, CMC; methacryloyl chitosan, MC acryloyl chitosan, MC) formed by a gel network of high crosslinking density linked by carbon–carbon double bonds simultaneously crosslinked with a catechol‐Fe^3+^ network. The design of this hydrogel with double cross‐linked dual network resulted in photocrosslinking properties, injectability, excellent toughness, tissue adhesion, excellent hemostatic properties, and antimicrobial activity, which also exhibited a better ability to promote wound healing in addition to a mouse model of infected wounds. It is worth to mention that the catechol‐Fe^3+^ chelating network of the hydrogel can be covalently linked with thiol, amino, imidazole, and other groups, which fundamentally enhances the adhesion of the gel to tissues. In the mechanical test, the lap shear strength of the gel to pigskin was 18.1 kPa, 5 times higher than that of commercially available and widely used fibrin glue (the gold standard of tissue adhesive).^[^ ^151^ ^]^ Mg^2+^ was reported to bind to bacterial cellulose after chelating with TA. This hydrogel released TA and Mg^2+^ uninterruptedly, and effectively inhibited the biofilm formation rate of *S. aureus* and *P. aeruginosa* by about 80% and 87%, respectively, after coculture with bacteria for 24 h.^[^ ^152^ ^]^

#### 4.1.2 Photothermal Antibacterial and Skin Regeneration

**Figure 8:**
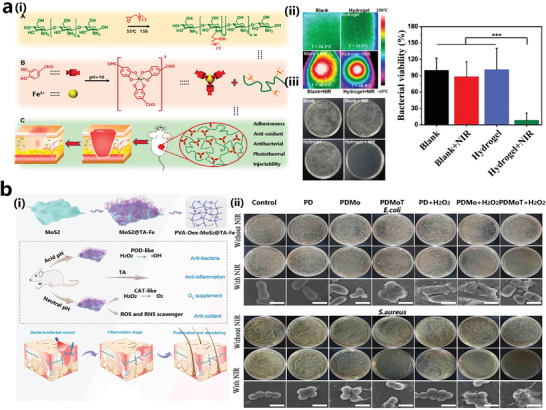
a, i) Dual dynamic cross‐linked QCSPA@Fe hydrogel consisting quaternary ammonium chitosan complexed with protocatechualdehyde (PA)‐Fe. ii) Temperature difference with and without quaternized chitosan protocatechualdehyde (QCSPA)@Fe hydrogel as shown by infrared thermal images and the bacterial plates after QCSPA@Fe hydrogel plus infrared treatment. Reproduced with permission.^[^ ^137^ ^]^ Copyright 2021, American Chemical Society. b, i) Schematic representation of the preparation of MoS2@TA/Fe‐PVA/Dex hydrogels with dual enzymatic activity and their promotion of skin repair and re‐epithelialization through photothermal antibacterial, oxygen release, anti‐inflammatory, antioxidant, and proangiogenic mechanisms. ii) Photographs and SEM images of bacterial colonies after hydrogel treatment plus NIR irradiation. Reproduced with permission.^[^ ^153^ ^]^ Copyright 2022, Elsevier.

**Figure 9:**
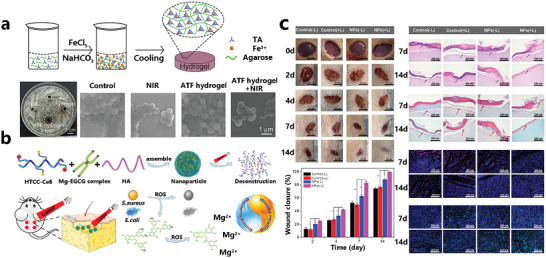
a) ATF hydrogel is composed of agarose, TA, and Fe^3+^ and its antibacterial properties. Reproduced with permission.^[^ ^154^ ^]^ Copyright 2019, Elsevier. b) A nanoparticle introduced with chlorin e6 (Ce6) photosensitizer and mainly composed of Mg‐EGCG‐HA complex with antibacterial properties. Reproduced with permission.^[^ ^155^ ^]^ Copyright 2019, American Chemical Society. c) Performance of Mg/EGCG nano complex to promote wound healing in infected rats and immunohistological staining of wound tissue (H & E, Masson, CD31, CD68). Reproduced with permission.^[^ ^155^ ^]^ Copyright 2019, American Chemical Society.

Photothermal therapy, a physical method that does not rely on antibiotics, has shown great potential as a new noninvasive technique for the treatment of bacteria and their biofilms. Its main principle is the conversion of light energy into heat to cause membrane disruption, protein deformation, and finally irreversible death of bacteria.^[^ ^156^ ^]^ Conventional inorganic photothermal materials such as various metal nanoparticles (Au, Ag, Cu), carbon‐based nanoparticles (graphene, carbon nanotubes), transition metal sulfides (CuS, WS2, MoS2), polymer‐based nanomaterials (polyaniline, poly(3,4‐ethylenedioxythiophene), polydopamine (PDA)), and organic small molecules (anthranilic dyes (indocyanine green, cypate, IR780 and IR825), prussian blue, black phosphorus, and red phosphorus) have been integrated into biomaterial systems and are widely used as photothermal antimicrobial agents.^[^ ^154^, ^156^, ^157^, ^158^ ^]^ Recently, scientists found that some multifunctional MPN composites also exhibit excellent photothermal properties. In a recent attempt, a smart hydrogel containing dual dynamic crosslinks, coordination between catechol and Fe^3+^, as well as reversible imine bonds (formed by Schiff base relation between protocatechuic aldehyde and quaternary ammonium chitosan (QCS)) was developed, exhibiting self‐healing, pH‐responsive, and on‐demand removable properties. Notably, this hydrogel showed a mild photothermal antimicrobial ability upon the irradiation of near‐infrared (NIR) light at 808 nm, and almost inhibited most of the pathogens at a power density of 1.4 W cm^−2^ and a irradiation time of 5–10 min, and its ability to heal *MRSA* infected animal wound was also demonstrated on an animal model (**Figure** **8**a).^[^ ^137^ ^]^ Molybdenum disulfide (MoS2), a transition metal sulfide, has been reported to have photothermal and peroxidase‐like nanoenzymatic properties. In Fan's work, TA‐Fe^3+^ modified MoS2 nanosheets were immobilized in a hydrogel network consisting of polyvinyl alcohol and dextran to confer the hydrogel with both favorable photothermal antimicrobial ability and peroxidase‐like enzymatic activity, which could inhibit almost 100% growth of *S. aureus* and *E. coli*. in vitro and promote the healing of infected wound healing in vivo (Figure 8b).^[^ ^153^ ^]^ Xu's group also developed TA‐Fe^3+^ compositing agarose hydrogel with excellent photothermal antimicrobial property, which could kill 99% bacteria assistant with NIR irradiation (**Figure** **9**a).^[^ ^154^ ^]^ Unlike photothermal therapy, photodynamic therapy is a method that uses photosensitizers to produce toxic ROS for the destruction of pathogens or cure of tumor. Wang used a photosensitizer chloride e6 (Ce6) coupled to quaternary ammonium chitosan and then compounded with Mg/EGCG coordination compound to prepare a multifunctional nanoparticle with synergistic chemical and light responsiveness, which could kill bacteria by producing ROS, at the same time EGCG was oxidized by ROS causing the release of Mg^2+^ from Mg/EGCG, thus synergistically promoted the healing of infected wounds (Figure 9b,c).^[^ ^155^ ^]^ Overall, in most of these studies, whether photothermal therapy (PTT) or photodynamic therapy (PDT) was utilized to combat wound bacterial infections, the increase in temperature and the production of toxic ROS were kept within a controlled range, avoiding excessive damage to the surrounding normal tissues. The strategy combining these strategies with biomaterials possesses the feasibility of applications involving microbial control of various infectious diseases (e.g., infected wounds, infected bone defects, periodontal infections, etc.), and the future research focusing in smart and even programmable control of temperature and ROS will be a promising area with potential applications.

### 4.2 Bone Regeneration

**Figure 10:**
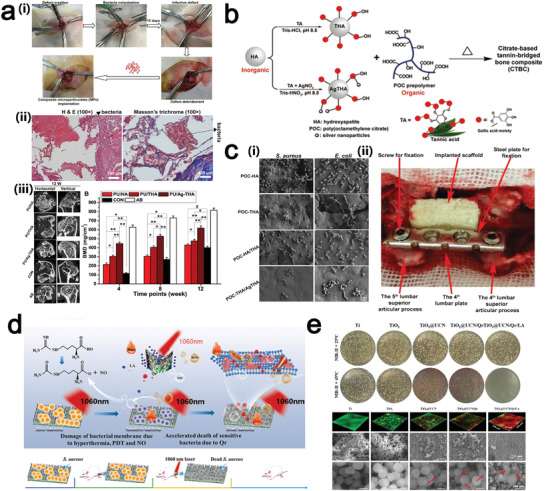
a, i) Application of PU/AgTHA composite microparticulates (MPs) in an infected femoral condylar defects animal model. ii) H&E and Masson‘s images of infected bone defects. iii) 2D CT images showing the effect of PU/AgTHA to the infected bone defects. Reproduced with permission.^[^ ^159^ ^]^ Copyright 2021, Elsevier. b) Citrate‐based tinnin‐bridged bone composites (CTBCs) fabricated by tannin and silver nanoparticle modified hydroxyapatite (AgTHA) covalently crosslinking with biodegradable citrate‐based bioelastomeric poly(octamethylene citrate) (POC). Reproduced with permission.^[^ ^160^ ^]^ Copyright 2020, Wiley‐VCH. c, i) The in vitro antimicrobial performance of CTBCs against *E.coli* and *S.aureus*. ii) CTBCs were used in a rabbit lumbar fusion model.^[^ ^160^ ^]^ Copyright 2018, Wiley‐VCH. d) An anti‐infection and osseointegration‐promoting coating consisting of La‐doped titanium dioxide/quercetin/L‐arginine (TiO2@UCN/QR/La) with rare earth elements was implanted into the tibia of mice and irradiated with a 1060 nm laser to combat microbial infection. Reproduced with permission.^[^ ^161^ ^]^ Copyright 2021, Elsevier. e) Photographs of bacterial colonies, bacterial live/dead images, and bacterial SEM images after TiO2@UCN/QR/La treatment. Reproduced with permission.^[^ ^161^ ^]^ Copyright 2021, Elsevier.

Bone defects caused by trauma, fracture, osteomyelitis, or bone tumor resection are often accompanied by infection, leading to poor clinical prognosis.^[^ ^162^, ^163^, ^164^, ^165^ ^]^ MPNs combine the specific functions of metal ions and polyphenol ligands, exhibiting unique advantages that are tailored to the desired properties of orthopedic biomaterials. Natural polyphenols contain plenty phenolic hydroxyl groups providing tissue adhesive, antioxidant, and anti‐inflammatory properties in addition to metal ions chelating ability, and some specific polyphenols have antimicrobial properties themselves. On the other hand, metal ions bring additional functions to MPNs, for example, Mg^2+^ has been reported to beneficial to osteogenesis and angiogenesis, Zn^2+^ has antibacterial, immunomodulatory, cellular metabolism‐regulating, and osteogenic properties, while Ca^2+^ is an important component of bone and involves in important physiological processes such as ion transport in osteogenesis and osteolysis, the introduction of these osteogenesis‐related metal ions is very important for bone regeneration.^[^ ^87^, ^166^ ^]^ MPNs can be involved in the preparation of bone composites as one of the important components of surface modification coatings or biomaterials, which are useful in the treatment of orthopedic diseases by their inherent intrinsic antibacterial ability or photothermal antimicrobial ability. Chen et al. prepared an Ag‐HA/TA composite coating by forming an array of anatase type titanium dioxide nanotubes on the surface of a titanium substrate through anodic oxidation and anneal, followed by TA coating and the deposit of silver nanoparticles reduced by TA in situ from Ag^+^. The coating exhibited strong antioxidant and antibacterial properties, and has promising applications in the field of oral implantation and bone formation.^[^ ^167^ ^]^ Our group has prepared tannic acid‐modified hydroxyapatite (THA) and tannic acid‐silver nanoparticle‐modified hydroxyapatite (Ag‐THA), respectively, by a simple one‐step method, and we compounded them with polyurethane (PU) to produce 250–425 µm composite microparticulates (MPs).The in vitro and in vivo antibacterial experiments demonstrated that PU/Ag‐THA exhibited superior antibacterial properties and the inhibition of bacterial growth benefited bone regeneration evidenced by the evaluated expression of osteogenesis‐related osteocalcin (OCN) and Runt‐related transcription factor 2 (RUNX2) as well higher bone mineral density in PU/Ag‐THA compared with other groups (**Figure** **10**a).^[^ ^159^ ^]^ Our group also used the same system (Ag‐THA) to composite with poly(octamethylene citrate) (POC) prepolymer to prepare citrate‐based tannin‐bridged bone composites (CTBCs) for lumbar fusion (Figure 10b), which exhibited favorable antimicrobial as well as enhanced osteoconductivity and osteoinductivity (Figure 10c).^[^ ^160^ ^]^ Zhang et al. constructed an array of titanium dioxide nanospade on titanium implants by hydrothermal reaction, followed by doping with ytterbium and erbium rare earth elements and covalently binding quercetin (Qr) under secondary hydrothermal and 1060 nm laser irradiation, respectively, and finally electrostatically adsorbing the negatively charged quercetin with the positively charged L‐arginine to form the final nanocomposite coating (Figure 10d). Notably, the coating can work simultaneously through PTT, PDT, NO release and quercetin's own effect. The rare earth elements Yb and Er are capable of generating reactive oxygen species upon irradiation with NIR‐II light. And the polyphenolic substance Qr as a flavonoid inhibited tumor growth, while producing antibacterial, anti‐inflammatory, and antiosteoporotic effects, which demonstrated the multifunctionality of MPNs (Figure 10e).^[^ ^161^ ^]^ Silibinin is an extract of the plant Silymarin (Artichoke), which belongs to the group of flavonoid lignans. It has been reported that its Zinc silibinin complex [Zn(sil)(H2O)2] formed by the coordination of silibinin with Zn^2+^, which can play a role in promoting bone growth through the specific miR‐590/smad7 signaling pathway. Not only that, but the complex also exhibited extraordinary provascular and antibacterial effects, which have potential application as a bone tissue engineering drug.^[^ ^168^ ^]^ In addition to antibacterial ability, many different MPNs such as TA/Ca^2+^, TA/Mg^2+^, EGCG/Mg^2+^, TA/Sr^2+^, and Kaemferal/Zn^2+^ exhibiting effective osteogenic property, thus suitable for the wide applications in bone regeneration.^[^ ^38^, ^87^, ^166^, ^169^, ^170^ ^]^

### 4.3 Anti‐Infection Coatings on Biomedical Devices

**Figure 11:**
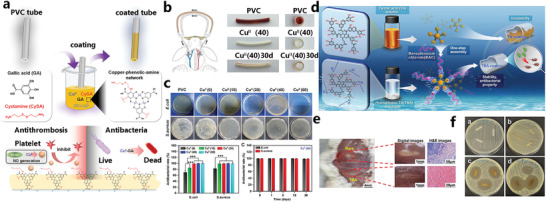
a) Cu^2+^‐GA/CySA coating deposited on polyvinyl chloride (PVC) medical catheters can be used to fight microorganisms and to induce the production of nitric oxide. b) Schematic diagram of a PVC medical catheter used for jugular arteriovenous shunts in New Zealand Rabbits. c) Antibacterial performance of Cu^2+^‐GA/CySA coated catheters. a–c) Reproduced with permission^[^ ^171^ ^]^ Copyright 2019, Royal Society of Chemistry. d) One‐step synthesis of hydrophobic benzalkonium chloride modified tannic acid (TBA) deposited on catheters by TA and benzalkonium chloride (BAC) has stability and antimicrobial properties. e) In vivo antimicrobial study, HE staining images, inflammation index, and bacterial colony counting of bare and TBA‐coated cannula in animals. f) The bacterial inhibition zone study of TBA coated catheter. d–f) Reproduced with permission.^[^ ^172^ ^]^ Copyright 2019, Royal Society of Chemistry.

Medical devices associated infections, such as suture infections, prosthetic valve infections, hernia repair mesh infections, syringe infections, catheter infections, and other related infections, have become a nonnegligible major medical problem. The risk of endocarditis (caused by infection) in patients with prosthetic heart valves implanted more than 20 years ago has been reported to be 7–15%. Catheter‐associated urinary tract infections (CAUTI) are the most common hospital‐acquired infections, with an average chance of 3.1–7.5 per 1000 catheter days. The infection rate for ventral hernia repair mesh fell in 1–10%. Bacteria are present in two main forms: 1) planktonic bacteria and 2) bacteria presenting in biofilms. The infections associated with medical devices are mainly caused by biofilms. Biofilms are composed of extracellular matrix secreted by the cells themselves, mainly composed of proteins, polysaccharides, DNA, RNA, and phospholipids, which are highly resistant to external stimuli.^[^ ^173^ ^]^ Currently, it is believed that bacterial biofilms are formed in four main stages: 1) adhesion, 2) accumulation, 3) maturation, and 4) detachment. In the last stage, the bacteria released from the biofilm will find new sites of adhesion and cause a wider range of secondary infections, which is the main reason why bacterial‐associated infections are difficult to completely eradicate once biofilms were formed.^[^ ^174^ ^]^ MPNs are likely to be a better choice than antibiotics as a novel antimicrobial agent for these difficult bacterial biofilm infections. Inspired by the surface chemistry of catecholamines and MPNs, Yang et al. developed a bifunctional coating consisting of gallic acid, Cu^2+^, and cystaminevia a facile one‐step impregnation (**Figure** **11**a). Cu^2+^ not only exerts its antithrombotic ability in synergy with gallic acid through its inherent antimicrobial properties, but also exhibits glutathione peroxidase (GPx) like activity to catalyze the production of NO from RSNO in blood (Figure 11b). In order to verify their antithrombotic ability, they were subjected to in vitro perfusion experiments on New Zealand Rabbits, and the lumen was barely occluded after 30 days test (Figure 11c).^[^ ^171^ ^]^ O‐phenylthriol (PG) and Ag^+^/Mg^2+^ were chelated and covered onto the inner and outer surfaces of the catheter, to reduce the hemolytic properties of silver without affecting its antimicrobial function, the coating can be also applied to other medical devices other than catheters (syringes, sutures, surgical instruments) or used in the development of antiseptic solutions and cleanrooms.^[^ ^175^ ^]^ Another antimicrobial coating for the prevention of catheter‐associated infections (CAI) was constructed by a one‐step assembly of TA and benzalkonium chloride (BAC) under alkaline conditions (pH 9) by electrostatic interaction (Figure 11d). The coating is colorless and transparent, has good biocompatibility and broad‐spectrum antimicrobial activity, and can be further enhanced by mixing with silver nitrate solution (Figure 11e,f).^[^ ^172^ ^]^

### 4.4 Other Fields

**Figure 12:**
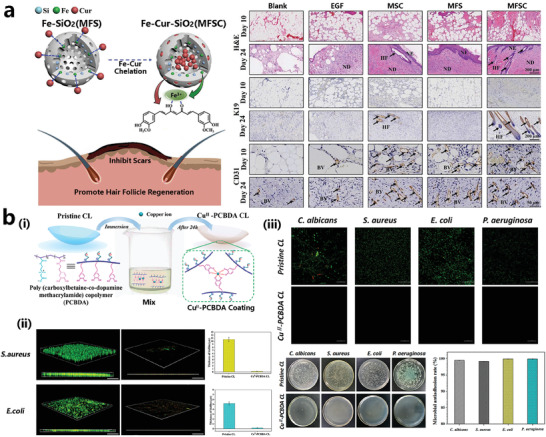
a) Schematic representation of the ability of curcumin/Fe‐SiO_2_ nanocomplex to inhibit scar regeneration and promote skin and hair follicle regeneration; the immunohistochemical staining of the skin and its appendages of mice are also presented. Reproduced with permission.^[^ ^176^ ^]^ Copyright 2021, Elsevier. b,i) Preparation of Cu^2+^‐poly(carboxylbetaine‐*co*‐dopamine methacrylamide) copolymer (PCBDA) coating on contact lens surface with antibacterial and antifouling properties. ii) The Live/Dead stained 3D images of *E. coli* and *S. aureus* attached to the contact lens surface detected by laser scanning confocal microscopy (LSCM) and the determined biofilm thickness. iii) The antibacterial performance of contact Cu^2+^‐PCBDA coated lenses against *C. albicans*, *S. aureus*, *E. coli*, and *P. Ae ruginosa*. Reproduced with permission.^[^ ^177^ ^]^ Copyright 2020, American Chemical Society.

**Figure 13:**
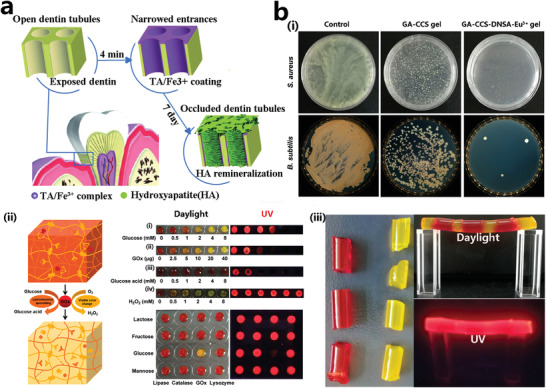
a) TA‐Fe^3+^ self‐assembled coatings were used to seal dentin microtubules on enamel to avoid tooth sensitivity and prevent potential bacterial infections.^[^ ^178^ ^]^ b, i) Antibacterial properties of GA/CCS/DNSA/Eu^3+^ hydrogels. ii) Schematic diagram of the sensing mechanism of GA/CCS/DNSA/Eu^3+^ hydrogels and the luminescent and self‐healing properties. iii) Fluorescence excitation properties of GA/CCS/DNSA/Eu^3+^ hydrogels. Reproduced with permission.^[^ ^179^ ^]^ Copyright 2020, American Chemical Society.

In addition to the antimicrobial applications in major fields such as skin wound repair, bone repair, and medical devices, MPNs have also been involved in scarless healing and hair regeneration, dental implants, and contact lenses coating, and even in wearable devices and smart sensing, where MPNs played antimicrobial roles and also served as components of flexible fabrics or gels.^[^ ^180^ ^]^ In the process of skin wound repair, the formation of scar is closely related to hair follicle regeneration. Chang designed a composite nanoparticle composing mesoporous silica/curcumin/Fe^3+^ with potential antibacterial ability, scar formation inhibition, and hair follicle promotion effects (**Figure** **12**a). Based on the starting point that long‐term contact lens (CL) wear may lead to eye dryness and CL‐related microbial keratitis (MK), Yu developed an MPN antimicrobial network consisting of Cu^2+^, and poly(carboxy betaine‐*co*‐dopamine‐methacrylamide) copolymer (PCBDA), where the catechol moiety and Cu^2+^ exhibited strong adhesion and broad‐spectrum antibacterial activity, respectively. It is an effective strategy to prevent corneal infections (Figure 12b).^[^ ^177^ ^]^ Although MPNs are less used in dentistry, TA, PG, and tea polyphenols have been reported to be used for antibiofilm infections in dental implants.^[^ ^181^ ^]^ The self‐assembled TA/Fe^3+^ films have been used to cover dentin tubules of human teeth, which can be effective in preventing tooth hypersensitivity as well as potentially infectious inflammation (**Figure** **13**a).^[^ ^178^ ^]^ In the field of smart materials, Wang et al. reported a multifunctional stimuli‐responsive luminescent GA/CCS/DNSA/Eu^3+^ hydrogel made from catechol‐modified carboxymethyl chitosan (CCS), phenylboronic acid‐modified gelatin (GA‐DBA), 3,5‐dinitrosalicylic acid (DNSA), and rare earth element Eu^3+^ ions after a simple heating‐cooling mixture. This GA/CCS/DNSA/Eu^3+^ hydrogel exhibited shape memory, self‐healing, information storage, naked‐eye sensing of glucose, and antimicrobial activity (Figure 13b), which showed reversible luminescence properties and phase change under the stimulation of temperature, salt, acid‐base, and redox, as well as excellent antibacterial effect against *Bacillus subtilis* and *S. aureus*.^[^ ^179^ ^]^

## 5 Conclusions and Outlook

In recent years, metal–phenolic networks (MPNs) have been increasingly used to combine with other substances and apply to skin wound healing, bone tissue repair, medical devices modification, and anti‐infection smart devices. However, the literature review on this part is sketchy or blank. Therefore, in this review, we focus on the antimicrobial applications of MPNs formed by the chelation of polyphenols with metal ions in the biomedical fields and outline the recent research progress. MPNs, as an amorphous chemical network of organic–inorganic hybridization, exist in various material forms (coatings, hydrogels, nanoparticles, capsules), and different forms of biomaterials play corresponding roles according to the different scenarios required for antibacterial purposes. At the same time, the preparation process of MPNs is simple and fast, green chemistry, and its most unique advantage is that the properties of polyphenols and metal ions themselves can be combined and then mutually modified or synergistically enhance each other's functions, with better performance and properties compared to single materials. Specifically in the antibacterial aspects of the performance of polyphenols and metal ions own inherent antibacterial properties, they work synergistically, the antibacterial effect is more obvious, while the good biocompatibility of natural polyphenols in a certain degree to reduce the biotoxicity of metal ions. Second, in some MPNs more unique photothermal effects to combat microorganisms. Overall, in the current context of bacterial resistance and antibiotic abuse in the medical field, this system has quite significant advantages in the control of microorganisms in the biomedical fields.

**Figure 14:**
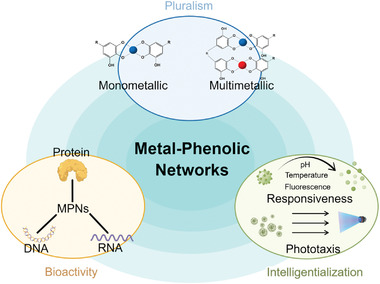
The future developing trends of MPNs.

Although the antibacterial application of MPNs in biomedical fields has achieved exciting results, it is still worthy of our consideration and investigation regarding the easy oxidation of polyphenols, the potential cytotoxicity of MPNs (too high concentration of polyphenols and metal ions, inappropriate ratio), and whether the photothermal antimicrobial or antimicrobial effect of MPNs and its biocompatibility can reach a balance or not.^[^ ^182^ ^]^ In addition to their outstanding performance in antibacterial, MPNs are expected to show great vitality in the future. At present, more and more works try to include binary or multicomponent metal ions rather than monometal in a single MPN (**Figure** **14**).^[^ ^71^, ^85^ ^]^ Compared with monometallic‐MPNs, multimetallic‐MPNs will be expected to exhibit more excellent multifunctional properties. In terms of bioactive biomaterials, polyphenols have been reported to be able to extensively combine with bioactive molecules (DNA, RNA, protein), and the biomaterials composed of MPNs and bioactive molecules is highly anticipated (Figure 14).^[^ ^183^, ^184^, ^185^ ^]^ Further, in the context of artificial intelligence, biomaterials with intelligent responsiveness and smart functions will attract much attention. In addition to the stimuli responsiveness (pH, temperature, fluorescence) that have been reported for MPNs, some MPNs structures have also been found to have unique phototaxis characteristics in recent years.^[^ ^186^ ^]^ MPNs materials incorporating the above‐mentioned smart functions have the potential to serve as biosensors or nanorobots in the future (Figure 14). With the further development of material science, we expect that biomaterials based on the MPNs will be designed more rationally and become a multifunctional platform suitable for advanced biomedical applications.

## Conflict of Interest

The authors declare no conflict of interest.
